# Polarized Light Microscopy for Quantitative Assessment of Sarcomere‐Scale Structural Order in Cardiac Tissue

**DOI:** 10.1002/jbio.70338

**Published:** 2026-08-10

**Authors:** Gennadii Piavchenko, Artem Venediktov, Zoya Shamitko, Karina Urazova, Mariia Obelchakova, Evelina Khabarova, Anton Ershov, Natalia Kartashkina, Viktoria Grikh, Valerii Smirnov, Galina Ramenskaya, Alexander Yatskovskiy, Igor Meglinski

**Affiliations:** ^1^ Human Anatomy and Histology Department I. M. Sechenov First Moscow State Medical University (Sechenov University) Moscow Russia; ^2^ Pathophysiology Department I. M. Sechenov First Moscow State Medical University (Sechenov University) Moscow Russia; ^3^ Analytical, Physical and Colloid Chemistry Department I. M. Sechenov First Moscow State Medical University (Sechenov University) Moscow Russia; ^4^ Pharmaceutical and Toxicological Chemistry Department I. M. Sechenov First Moscow State Medical University (Sechenov University) Moscow Russia; ^5^ College of Engineering and Physical Sciences Aston University Birmingham UK

**Keywords:** cardiac arrest, histopathology, myocardial ischemia, polarized light microscopy (PLM), respiratory arrest, sarcomere structure

## Abstract

Polarization‐resolved imaging provides a powerful, label‐free means of visualizing anisotropic structural order in biological tissue, yet its quantitative exploitation remains limited in routine microscopy. Here, we present a polarization‐resolved imaging approach based on polarized light microscopy (PLM) that enables automated, quantitative extraction of sarcomere‐scale structural metrics from unstained cardiac tissue sections. Using experimental rat models of acute cardiorespiratory arrest as controlled test systems, we demonstrate that polarization‐derived intensity profiles encode reproducible information on sarcomere length and band composition, with distinct patterns of structural compression observed across experimental conditions. Image‐derived measurements were validated against conventional histological and immunohistochemical staining, highlighting the complementary value of polarization contrast for rapid, staining‐free assessment of tissue anisotropy. Rather than addressing diagnostic specificity, this proof‐of‐concept study establishes PLM as a scalable quantitative imaging modality for polarization‐resolved analysis of hierarchical structural order in biological tissue, with potential integration into multimodal imaging workflows in biomedical research.

## Graphical abstract

Impact of cardiac and respiratory arrest on cardiac muscle tissue. Wistar rats were injected (*on the left*) either with cisatracurium to induce a respiratory arrest or lidocaine to induce a cardiac arrest (*in the middle*). Further, the pathophysiological changes developed and were assessed with linear polarization microscopy at 600–3000 nm wavelength range (*on the right*), enabling quantitative, rapid, and staining‐free distinguishing between ischemic damage caused by cardiac and respiratory arrests.

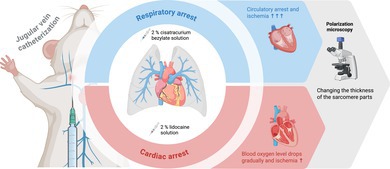

## 1 Introduction

Studying histological sections with polarized light can be informative to reveal structural features of tissue samples without need for electron microscopy, dyes, or fluorescent labelling [1]. In contrast to conventional histochemical and immunohistochemical (IHC) methods, the microscopy of unstained slides, including polarizing light microscopy, may lose in the number of structures revealed but also win in the list of optical properties visualized in biological tissues [2, 3]. Exactly, some modifications of light waves may result in a visible contrast between objects observed, thus making it possible to examine both native specimens and unstained fixed tissue sections [4].

Polarized light microscopy (PLM) was first invented by Talbot in the XIX century [5] and entered common medical research in the early 60s [6], but its clinical application remains limited. In biological tissue, the propagation of polarized light depends on the refractive index, as well as tissue scattering and absorption properties [7]. When the refractive index is heterogeneous, the beams of polarized light pass through the tissue and split into waves with different refraction, propagation velocity, and plane of polarization; this effect is called birefringence [8]. Birefringent objects are anisotropic due to the arrangement of molecules and fibrillar structures [7]. However, the level of birefringence can change in tissue stretching, compression, or exposure to electromagnetic fields changing the location of molecules or atomic orbitals [8].

PLM or its modification, Mueller polarimetry, are therefore useful to identify anisotropic structures [9, 10], including those that emerge in disease. For instance, it may be appropriate for assessment of ischemic myocardial damage [2, 11]. As a matter of fact, cells of cardiac muscle tissue in the myocardium, cardiomyocytes, contain myofibrils consisting of actin and myosin myofilaments. Briefly, their colocalization may be described as parallel cylinders. Longitudinal sections and cross‐sections of the cylinders share different values of refractive index, both not equal to the refractive index in neighboring tissues. Alongside the cylinders, some portions are anisotropic because of heavy proteins, actin and myosin together (A‐bands from “anisotropic”), whereas others are isotropic with light proteins, actin almost only (I‐bands from “isotropic”). Together, an A‐band and two neighboring halves of I‐bands constitute a functional unit of the tissue, a sarcomere. Thus, cardiac muscle tissue is birefringent media with alternation of anisotropic and isotropic bands, and this is accessible for detection with polarizing light microscopy [7, 12].

**FIGURE 1:**
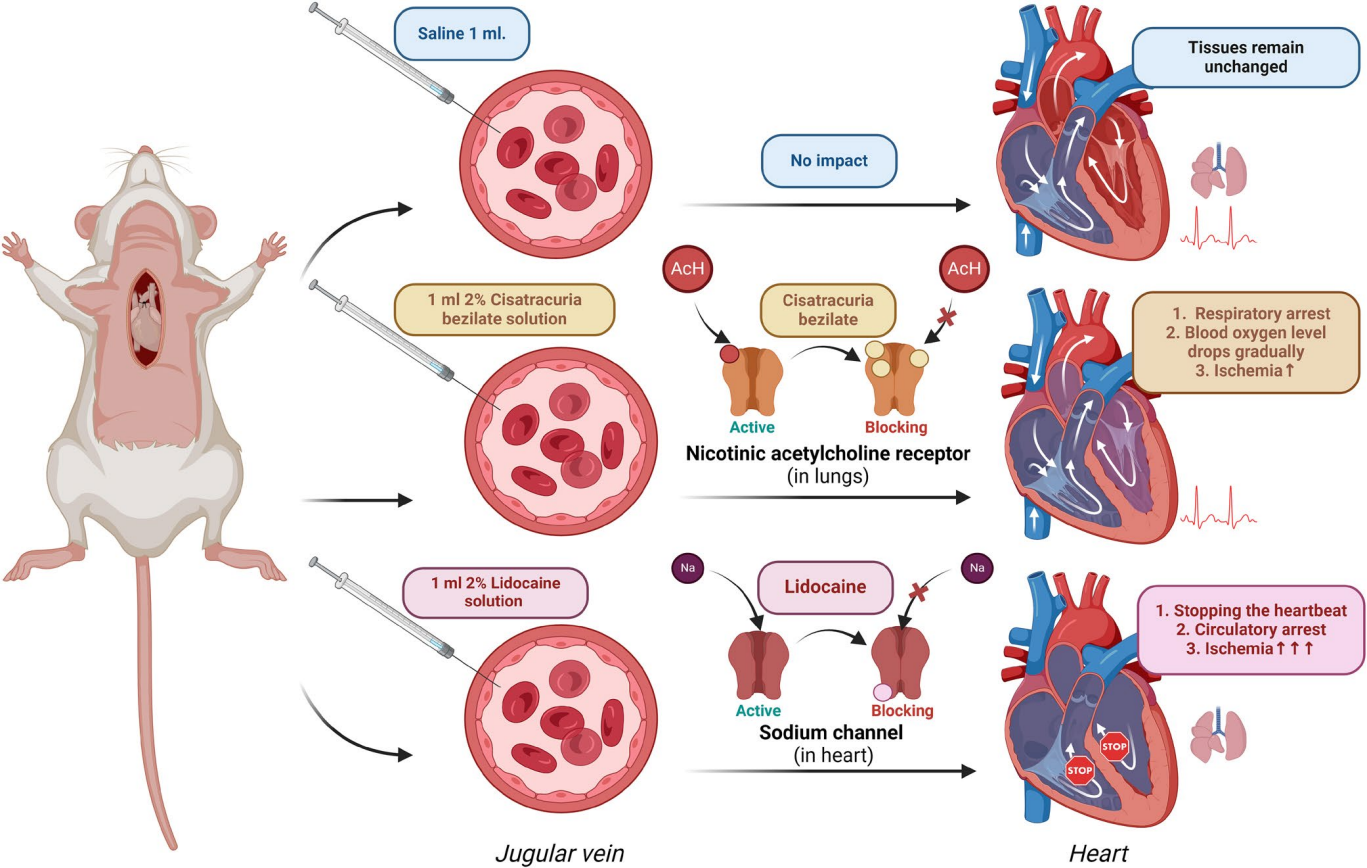
Design and principles of the study. To observe changes in the structure of sarcomeres, we regarded the control group and two experimental groups with acute respiratory or cardiac arrest modeled. Pathophysiological changes after cisatracurium and lidocaine actions are shown.

Cardiomyocytes are long‐living cells that have actually no appropriate source of regeneration, and due to this any impairment in their oxidative metabolism can be fatal [13] (Figure 1). For instance, hematoxylin and eosin staining of ischemic (i.e., oxygen deprived) cardiomyocytes reveals their eosinophilic infiltration, destruction of myofibrils and nuclei, and their undular geometrical arrangement [14]. A long period of myocardial ischemia even provokes irreversible structural changes at electron microscopy, such as autophagic vacuoles, swollen mitochondria, deformed or missing cristae in them, destruction of myofilaments, and enlargement of sarcoplasmic reticulum [15, 16]. Some sources also report gaps in plasma membrane and detachment of cytoskeletal elements from mitochondria [17, 18]. In myocardial ischemia–reperfusion injury, reactive oxygen species and lipid peroxidation products bring additional damage to cardiomyocytes [19, 20]. Besides, sarcoplasmic reticulum stress, calcium overload, and lack of ATP favor the development of apoptosis, ferroptosis, and autophagy [21, 22, 23, 24, 25]. Myocardial ischemia–reperfusion injury can be also accompanied by neutrophilic infiltration of the tissue, as well as by a decreased mitochondrial volume and disintegrity of mitochondrial membranes in cardiomyocytes [17, 24, 25].

Therefore, ischemic tissue of myocardium obtains changes in myofibrils, and that is accessible by PLM [26]. As myofibrils experience excessive contraction, I‐bands should decrease, and polarized light reveals a resulting increase in anisotropy up to a convergence of A‐bands or even a complete fusion into a continuous anisotropic conglomerate [27]. Besides, long‐time oxygen‐deprived myofibrils undergo both disintegration and myocytolysis in PLM, when anisotropic clusters of transformed sarcomeres alternate with isotropic foci of uncompressed destroyed myofibrils [28]. Such damage marks apoptosis initiation and autophagy with the activation of hydrolytic enzymes [21, 22, 29].

However, many cases of non‐fatal myocardial ischemia are of short‐term, concerning either acute cardiac or respiratory arrest, although resulting in different degrees of myocardial damage [30]. We assume if these well‐known differences in the degree of myocardial damage are accurately distinguished by PLM at unstained slides. Therefore, our study aimed to assess the myocardial damage with PLM in two basically different acute conditions related to oxygen shortage, namely cardiac and respiratory arrest, and to compare this assessment to conventional methods of cardiac muscle tissue visualization in histological studies.

## 2 Materials and Methods

### 2.1 Animal Care

Our study included nine male Wistar rats obtained from the Stolbovaya branch of the Federal State Budgetary Institution “Scientific Center for Biomedical Technologies” of the FMBA of Russia. The animals included in the experiment were randomized into three groups (*n* = 3). The rats had a mean value of body mass equal to 150 ± 3.5 g at the beginning of this experimental study. All the design and manipulations performed satisfied requirements and principles of Good Laboratory practice. The study was approved by the Local Ethical Committee of Sechenov University (Protocol No. 04‐23 of 03/02/2023).

### 2.2 Cardiac and Respiratory Arrest Modeling

To perform a histological study of myocardial structure, we initially catheterized the right external jugular veins of the animals with the following cardiac arrest by administration of 1 mL of 2% lidocaine solution (Experimental Group 1) or respiratory arrest by administration of 1 mL of 2% cisatracurium bezylate solution (experimental Group 2) (Figure 1). To support the vital functions of the heart, we performed additional adrenaline injections into the central bloodstream. Group 3 was a control one with sham intervention (saline solution only).

### 2.3 Histological Study

Alive animals underwent fixation by administration of neutral buffered formalin into the systemic circulation (left ventricle). Immediately after that, an autopsy was performed with the heart extraction and immersion into formalin. We performed 5 μm histological sections of the myocardium and used them to study the anisotropy of cardiomyocytes after deparaffinization (although the deparaffinization buffer solutions may affect the results, paraffins are birefringent, and we preferred to remove them to reduce a possible bias). Histological sections were stained by either hematoxylin and eosin (H&E), Weigert's iron hematoxylin, Mallory's trichrome, or Lie's/hematoxylin–basic fuchsin–picric acid (HBFP) methods (*n* = 6 per technique), whereas six sections from any animal were not stained after xylene deparaffinization to analyze them with a transmitted polarizing microscope. Besides, we provided IHC staining of Ki67^+^ cells using rabbit polyclonal antibodies (1:15 000; clone SR00‐02, No. HA721115, lot HP0304; Huabio, PRC) and 3′3‐diaminobenzidine chromogen.

### 2.4 Image Analysis

We employed linear PLM method to study images in the working range of polarizer at 480–550 nm (Thorlabs, USA), object lens ×40 (numerical aperture 0.75), and Axiocam 305 color camera (Zeiss, Germany). Circular and Mueller matrix polarimetry were avoided as no data on chiral structures was required for the study needs.

We have obtained microphotographs of both stained and unstained sections with Zen 3.10 software (Zeiss, Germany). For unstained sections, we further calculated the lengths of whole sarcomere, A‐bands, and I‐bands. The calculations for PLM were provided with our own Python‐based tool (version 3.12.4, Python Software Foundation, USA) to determine the color of pixels along a given straight line. For Ki67‐staining, we counted the number of positive cells per field of view at the total magnification of ×400 for six slides per animal.

### 2.5 Statistics and Reproducibility

Statistical analysis of the data was performed with Origin Pro software (OriginLab, USA). After distribution normality Shapiro–Wilk test, we used parametric (ANOVA with Tukey post hoc comparison) and non‐parametric methods (Kruskal–Wallis with Dunn post hoc comparison) to compare the samples. The sample size of three animals per group was considered significant at a *p* value less than 0.05 and statistical power more than 0.8 with six sections per animal for any staining and 12 fields of view analyzed for a section. The sample size is appropriate for the proof‐of‐concept study design, especially with respect to the number of fields of view analyzed.

## 3 Results and Discussion

### 3.1 Quantitative PLM Assessment of Sarcomere Structure

**FIGURE 2:**
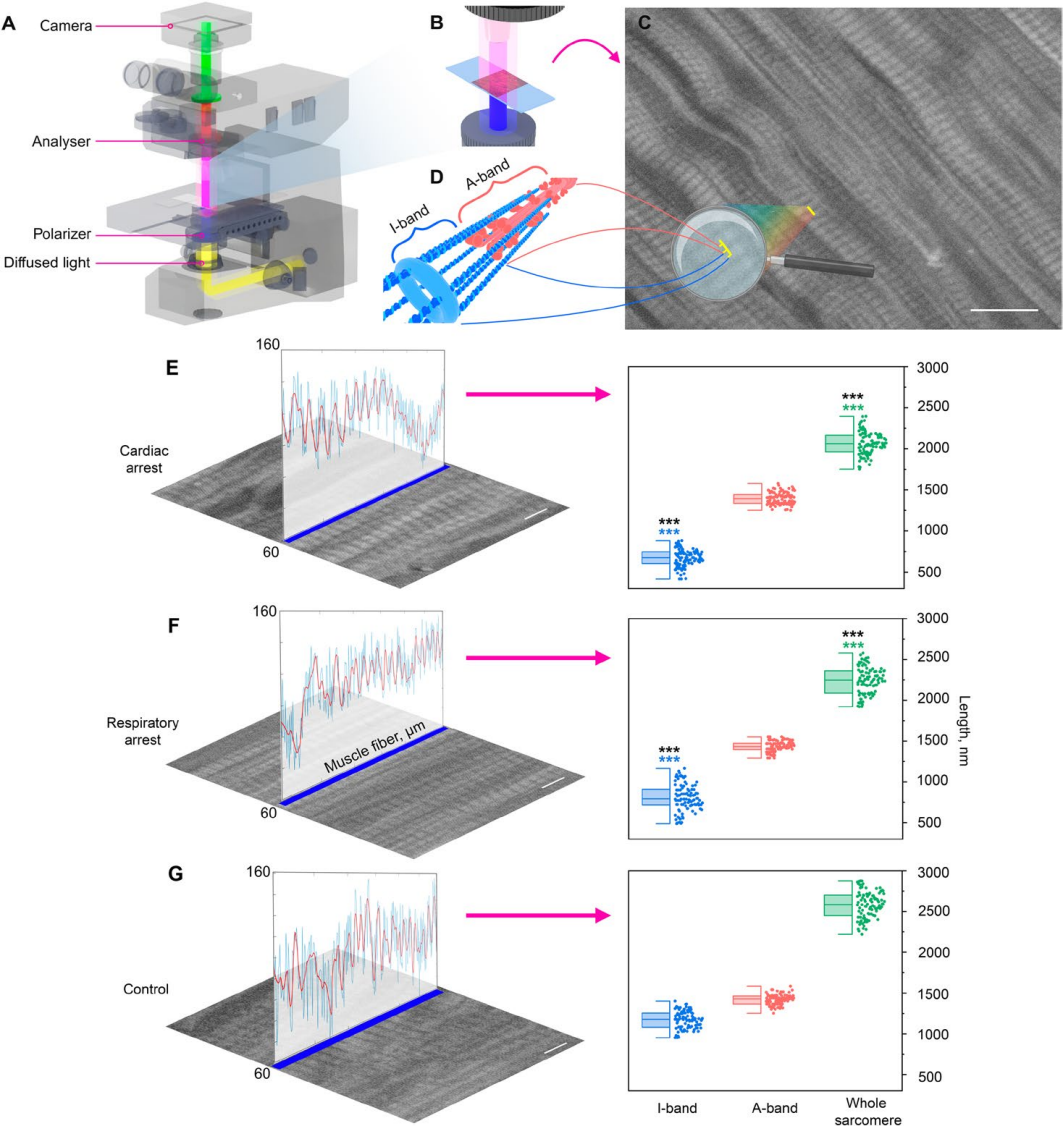
Polarizing light microscopy (PLM) for assessment of quantitative sarcomere values. (A) Schematic representation of PLM, light passing through cross‐polarizer with a specimen and then either to camera or ocular lens. (B) Specimen of unstained slide to reveal (C) cardiac muscle tissue with separate sarcomeres (green; scalebar 20 μm). Adjusting a larger magnification at the microscope (lower left corner, scalebar 2 μm), one can better see A‐bands of sarcomeres looking like light lines and I‐bands of sarcomeres looking like dark lines, what is graphically modelled at (D). In damage, the ratio between A‐ and I‐bands changes, as well as the total sarcomere length. To assess this, we measured segments of the same length in cardiac arrest (E), respiratory arrest (F), and intact animals (G) (scalebar 5 μm), then transforming these segments into plots (above blue pixel lines) showing the intensity of polarized light (*Y* axis) alongside the segments (*X* axis). Values of *X* axis below the moving average are I‐bands, and above are A‐bands, and each period of intensity‐to‐length function is a whole sarcomere. Counting the average length of I‐bands, A‐bands, and whole sarcomeres, we obtained the results represented on plots where color asterisks mark the difference between samples with cardiac and respiratory arrest, black asterisks, to the control group. ***p < 0.05.

Unstained deparaffinized sections of myocardium, split into distinct regions, were subsequently subjected to software processing. We measured sarcomere parameters in gray‐scale for three groups, plotting the intensity of the color indicator (Figure 2). Length difference for sarcomeres and their parts determined, we obtained a comparative graph for the studied parameters, as well as separate graphs for sarcomeres, A‐ and I‐bands, respectively.

Our study of the polarization properties in cardiomyocytes showed that sarcomere length decreases significantly in both respiratory arrest and cardiac arrest. The average sarcomere length was equal to 2582.2 ± 16.12 nm in animals of the control group (95% CI: 2550.2–2614.2 nm), at respiratory arrest it reached 2242.8 ± 16.47 nm (95% CI: 2210.1–2275.5 nm), and at cardiac arrest—2065.5 ± 13.65 nm (95% CI: 2038.4–2092.6 nm).

The size of I‐bands also decreased in the experimental groups. Thus, the average length of I‐bands in the control group stayed as 1165.3 ± 11.02 nm (95% CI: 1143.4–1187.2 nm), for respiratory arrest—811.2 ± 16.43 nm (95% CI: 778.6–843.8 nm), for cardiac arrest it decreased to 672.4 ± 10.70 nm (95% CI: 651.1–693.6 nm). At the same time, the median values for A‐bands in all groups did not differ significantly, and had a non‐Gaussian distribution.

### 3.2 Comparison With Conventional Histological Methods

**FIGURE 3:**
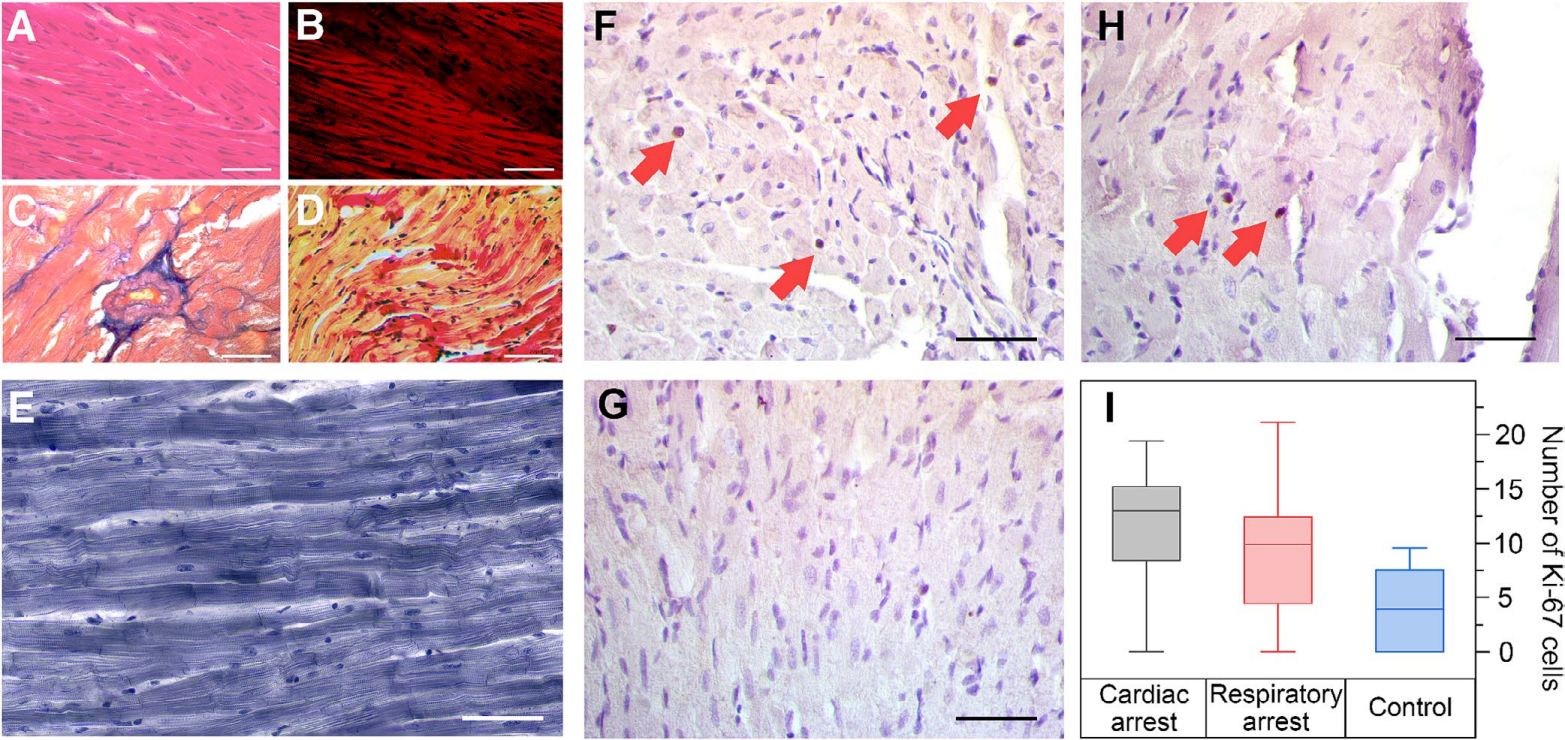
Histological study of stained slides: Ischemic myocardium in rats. (A) Light microscopy with hematoxylin and eosin (H&E) staining, ×200; (B) the same section, polarized light, intense anisotropic areas are visible in some cardiomyocytes, ×200; (C) Mallory's trichrome staining, ×200; (D) Lie staining (HBFP), some areas of early ischemia are visible, ×200; (E) Weigert's iron hematoxylin staining, the striation of cardiac muscle tissue is visible, ×800; (F–H) groups with cardiac arrest, sham intervention, and respiratory arrest, respectively. Ob. ×40. Red arrows mark the Ki67+ cells. Scalebar 40 μm; (I) the number of Ki67^+^ cells.

Besides, we provided a histological study by conventional methods with stained slides to compare the visualization with our unstained sections. We employed routine and histochemical reactions to identify ischemic areas and other morphological signs of hypoxic tissue damage. At H&E‐stained slides (see Figure 3A), few changes are seen, although their visualization in polarized light reveals strong anisotropy in some sites (see Figure 3B). Histochemical stains provide more relevant qualitative information; exactly, Mallory's staining and Lie's staining are commonly known for several decades to assess the tinctorial features of ischemic zones [31, 32]. Thus, at Mallory's trichrome staining (see Figure 3C), ischemic zones obtain orange to yellowish color [31]. Besides, connective tissue can be easily distinguished due to its bluish color. A better understanding of ischemia localization is achieved at Lie's staining (HBFP) [32]. Here, fuchsinophilic damaged cardiac muscle cells can be observed even in early ischemia, the sarcoplasm of such cells stains bright purple‐red, whereas intact cardiomyocytes maintain yellow color (see Figure 3D). Nevertheless, all these methods, except the combination with polarized light at Figure 3B, permit no easy‐to‐access measuring of sarcomere structure. For this, Weigert's iron hematoxylin staining is employed, revealing striated elements of cardiac muscle tissue and intercalated discs (see Figure 3E), although this method requires relatively long timing.

IHC staining has also been provided to check if there exists mitotic activity after the ischemia. According to the results of IHC staining for Ki67^+^ antibodies, we observe that some cells are able to re‐enter the cell division cycle (Figure 3F–I). Meanwhile, the number of proliferating cells in the myocardium is significantly higher in cardiac arrest compared to the respiratory one, implying a more prominent reaction to the damage. In the control group at Figure 3G, one can see no antibody reaction to Ki67, as no damage has been caused and no proliferation resulted therefore.

### 3.3 General Discussion

Classical histochemical approaches have a limited application despite being used for more than a century. For instance, in urgent cases, both routine histological and histochemical visualization require a relatively large period of time to accomplish the staining. Besides, there is a challenge to avoid an alteration brought to the native structure of tissue by chemical dyes. This alteration of the native structure also involves a possible bias at quantitative assessment, despite the absolute indispensability of histochemical staining for qualitative study. IHC methods escape this disadvantage, being useful for quantitative research as well as for qualitative one. However, any IHC staining requires even more time than common histochemical reactions. IHC approach is more expensive, and its implementation comprises additional negative and positive control slides. Moreover, the number of IHC markers that can be detected on a single slide is very limited.

In myofibrils, alternating light and dark bands constitute striations, whose profile can be represented as a sinusoidal curve. The sinusoid frequency, which reflects the distance between the dark bands (A‐bands) and therefore the length of the sarcomere, can be extracted by the analysis of Fourier spectrum for the corresponding cardiomyocyte image [33, 34, 35, 36]. In addition to this method, a simple calculation of the number of bands is also appropriate to measure sarcomeres, followed by dividing a sarcomere's length by the number of bands [37]. Besides, both light microscopy and transmission electron microscopy (TEM) are applicable for the sarcomere length measurement, as well as PLM does [38]. It should be noted that PLM does not visualize individual molecules directly, but rather captures optical signatures arising from the ordered organization of actin–myosin filaments and the contraction state of sarcomeres, thereby providing structural readouts that reflect underlying molecular processes.

Usually, the length of sarcomeres in human and rodent cardiomyocytes (according to various sources) is equal to approximately 2.0–2.3 μm when relaxed and approximately 1.5 μm when contracted [33, 34, 38, 39, 40], and this is consistent with our data. Some authors report [37] an increase of cardiomyocyte sarcomere length during ongoing myocardial ischemia. Excessive contraction of sarcomeres is one of the earliest and most typical morphological features of ischemic myocardium, whereas the sarcomere length decreases to less than 1.5 μm.

Obviously, acute hypoxia in cardiomyocytes and a subsequent decrease in ATP content contribute largely to the development of this excessive contraction. Without ATP, there is no phosphorylation of myosin light chains by myosin light chain kinase—calcitonin related polypeptide alpha complex, and no relaxation is allowed. In addition to the excessive contractions, some segments of sarcomeres may experience opposite activity, stretching above 2.2 μm. This effect is mediated by a decrease in the levels of calcium ions in the sarcolemma due to the continuous membrane repolarization as a result of blocked potential‐dependent dihydropyridine channels (L‐type Ca^2+^ channels), which are found in the sarcolemma, especially in its system of T tubules. When the transport of Ca^2+^ ions from outside the cell is blocked, the release of Ca^2+^ from the sarcoplasmic reticulum is also disabled, since calcium‐sensitive ryanodine receptors get into an inactive state due to a decreasing content of calcium ions in the sarcoplasm [41].

One of the modern methods to measure the length of sarcomeres, concurrent to PLM, is second‐harmonic imaging microscopy [42]. It uses an effect of second harmonic generation (SHG), a second‐order nonlinear optical process that occurs in birefringent crystals or in non‐centrosymmetric biological structures such as collagen, myosin filaments, and microtubules [43]. In SHG, two photons of the same wavelength coalesce into a virtual state to constitute a single photon with an energy exactly twice the energy of the original photons. Moreover, the resulting wavelength is half as long as the original one [44]. The second harmonic photon is generated almost instantly (within a few femtoseconds), so that the second harmonic signal is coherent and radiates predominantly in a straight direction [45]. This method enables obtaining contrast images of sarcomeres at unstained histological sections with clearly distinguishable A‐bands, so calculations get simpler [46, 47]. Other similar studies were previously performed with concurrent approaches. Thus, Lu et al. [38] investigated the myocardium in pigs after a 90‐min ischemic period with left ventricle blood flow at 29% ± 7% of the initial level, followed by a 90‐min reperfusion. There, TEM revealed that the average length of sarcomeres after ischemia with subsequent reperfusion (2.33 μm) was higher than in ischemia without reperfusion (2.27 μm) and in animals of the control group (2.14 μm) (consistent with our data). The authors outlined that morphometrics showed the transverse distance between myofibrils to be less in ischemic areas of the myocardium than in non‐ischemic ones.

Another study regarded [40] the length of sarcomeres achieved from human‐induced pluripotent stem cells (hiPSC) compared to neonatal and adult human cardiomyocytes. The authors reported using fluorescence microscopy with α‐actin labelling. This study revealed that the average length of sarcomeres obtained from hiPSC was 1.84 μm. The same results were present for neonatal cardiomyocytes, and it was almost the same with adult cardiomyocytes (1.91 μm). Thus, the immature phenotype of hiPSC‐derived cardiomyocytes limited their potential for cell therapy. Currently, no cardiomyocytes similar to adult ones are derived from hiPSC stem cells.

Additionally, a study using the SHG method with 20 nm resolution employed alive unstained cardiomyocytes from frogs [47]. The study evaluated the effects of saxitoxin (blocks potential‐dependent Na^+^ channels) on the length of cardiomyocyte sarcomeres. Normally, the length of sarcomeres was 2.31 μm, and it reached 2.14 μm after the treatment (consistent with our data). The authors remarked that the length reduction was mainly due to the length of I‐bands.

All these approaches from studies cited, exactly TEM, fluorescent microscopy or spectroscopy [48], and SHG, take more time than PLM and/or are more expensive. Nevertheless, none of them, as well as PLM, is widely used in medicine. Main clinical methods to assess ischemic myocardial damage include routine hematoxylin–eosin staining and IHC reactions [49, 50, 51]. The most common antibodies here are plasma markers such as C5b‐9 and fibronectin, connexin 43, cardiac troponin I, and myoglobin [52]. Ki67 antibody is also used to identify cells that re‐enter the cell‐division cycle [53].

Our findings position PLM as a complementary and disruptive alternative to established techniques for assessing myocardial structure. TEM remains the gold standard for visualizing sarcomere organization at nanometer resolution, yet it is time‐consuming, technically demanding, and unsuitable for rapid diagnostics. X‐ray diffraction has historically provided precise sarcomere length measurements, but required synchrotron facilities and lacked applicability in routine pathology. SHG and multiphoton imaging offer label‐free assessment of myofibrillar architecture with high resolution, but these approaches demand expensive laser systems and complex instrumentation. By contrast, PLM enables rapid, staining‐free quantification of sarcomere length and band composition directly from unstained tissue sections using accessible optical hardware [54]. While not delivering molecular‐level resolution, PLM captures optical signatures of actin–myosin alignment that closely reflect underlying molecular processes, providing quantitative results consistent with values reported by TEM and SHG studies. These features make PLM uniquely suited for translation into routine histopathology, emergency diagnostics, and forensic workflows.

To sum up, the current proof‐of‐concept study shows that PLM enables quantitative, rapid, and staining‐free detection of myocardial ischemic damage. By distinguishing between cardiac and respiratory arrest, PLM holds promise as a translational tool for emergency medicine, forensic pathology, and future clinical cardiology.

Study limitations. The study employed one PLM method only, and a comparative study with more device options would provide additional insight. However, we aimed to show the proof‐of‐concept modality rather than provide an overall comparison. Further, both cardiac and respiratory arrest animal models are for sure not equal to the relevant processes in the human body. Nevertheless, lidocaine and cisatracurium for animal modeling of cardiac and respiratory arrest, correspondingly, are commonly known for a long time [55, 56, 57].

## 4 Conclusion

This study demonstrates that polarization‐resolved light microscopy enables rapid, quantitative, and label‐free imaging of sarcomere‐scale structural order in biological tissue. By extracting image‐derived metrics from polarization‐dependent intensity profiles, PLM provides reproducible measures of anisotropic organization that are consistent with values obtained using more complex and resource‐intensive imaging techniques. The experimental models employed here serve as controlled test systems to evaluate imaging sensitivity to structural compression states, rather than to establish diagnostic specificity or mechanistic attribution.

While PLM does not resolve molecular‐scale features directly, it captures robust optical signatures arising from the ordered arrangement of actin–myosin assemblies, offering a practical balance between physical interpretability, accessibility, and throughput. These characteristics position PLM as a valuable quantitative imaging tool for studying hierarchical anisotropy in biological materials and for complementing existing structural imaging modalities. Future extensions incorporating full polarimetric measurements, advanced computational analysis, or multimodal imaging integration may further enhance the utility of polarization‐resolved approaches for quantitative biomedical imaging.

## Author Contributions

G.P. and I.M. developed the concept. K.U., M.O., and E.K. performed the experiments. A.Y., G.P., N.K., A.E., V.G., V.S., G.R., and I.M. performed data analysis. Z.S., A.V., A.Y., and N.K. wrote the original manuscript. G.P. and I.M. edited the final version of the manuscript and supervised the experiments. All authors reviewed the manuscript and agreed with the submitted version.

## Funding

Authors acknowledge the support from the European Union’s Horizon Europe Research and Innovation Programme, OPTIPATH project, Grant Agreement No. 101185769.

## Ethics Statement

All animal experiments were conducted in accordance with Good Laboratory Practice requirements and principles. The study was approved by the Local Ethical Committee of I. M. Sechenov First Moscow State Medical University (Sechenov University) (Protocol No. 04‐23 of 03/02/2023).

## Conflicts of Interest

The authors declare no conflicts of interest.

## Data Availability

The data that support the findings of this study are available from the corresponding author upon reasonable request.

## References

[1] A. Keikhosravi , M. Shribak , M. W. Conklin , et al., “Real‐Time Polarization Microscopy of Fibrillar Collagen in Histopathology,” Scientific Reports 11, no. 1 (2021): 19063. 34561546 10.1038/s41598-021-98600-w PMC8463693

[2] V. Bachinsky , O. Vanchulyak , A. Ushenko , et al., “Diagnosis of Acute Coronary Insufficiency by the Method of Mueller Matrix Analysis of Myosin Myocardium Networks,” in Multi‐Parameter Mueller Matrix Microscopy for the Expert Assessment of Acute Myocardium Ischemia (Springer Singapore, 2021), 53–78.

[3] C. He , H. He , J. Chang , B. Chen , H. Ma , and M. J. Booth , “Polarisation Optics for Biomedical and Clinical Applications: A Review,” Light: Science & Applications 10 (2021): 194. 10.1038/s41377-021-00639-x PMC8458371 34552045

[4] T. Liu , K. de Haan , B. Bai , et al., “Deep Learning‐Based Holographic Polarization Microscopy,” ACS Photonics 7, no. 11 (2020): 3023–3034. 34368395 10.1021/acsphotonics.0c01051 PMC8345334

[5] H. F. Talbot , “Experiments on Light,” London, Edinburgh, and Dublin Philosophical Magazine and Journal of Science 5, no. 29 (1834): 321–334.

[6] D. Carlstroem , “Polarization Microscopy of Dental Enamel With Reference to Incipient Carious Lesions,” in Advances in Oral Biology, vol. 1, ed. P. H. Staple (Academic Press, 1964), 255–296. 14247278

[7] V. V. Tuchin , L. V. Wang , and D. A. Zimnyakov , “Optical Polarization in Biomedical Applications,” in Optical Polarization in Biomedical Applications (Springer Berlin Heidelberg, 2006).

[8] S. Inoué , “Polarization Microscopy,” in Current Protocols in Cell Biology (New York: John Wiley, 2002). 10.1002/0471143030.cb0409s13 18228405

[9] M. Kim , H. R. Lee , R. Ossikovski , A. Malfait‐Jobart , D. Lamarque , and T. Novikova , “Optical Diagnosis of Gastric Tissue Biopsies With Mueller Microscopy and Statistical Analysis,” Journal of the European Optical Society‐Rapid Publications 18 (2022): 10.

[10] A. Sdobnov , V. Ushenko , L. Tryfonyuk , et al., “Mueller‐Matrix Imaging Polarimetry Elevated by Wavelet Decomposition and Polarization‐Singular Processing for Analysis of Specific Cancerous Tissue Pathology,” Journal of Biomedical Optics 28, no. 10 (2023): 102903. 37425430 10.1117/1.JBO.28.10.102903 PMC10329407

[11] M. F. Wood , N. Ghosh , M. A. Wallenburg , et al., “Polarization Birefringence Measurements for Characterizing the Myocardium, Including Healthy, Infarcted, and Stem‐Cell‐Regenerated Tissues,” Journal of Biomedical Optics 15, no. 4 (2010): 047009. 20799840 10.1117/1.3469844

[12] C. M. Risi , I. Pepper , B. Belknap , et al., “The Structure of the Native Cardiac Thin Filament at Systolic Ca2+ Levels,” Proceedings of the National Academy of Sciences of the United States of America 118 (2021): e2024288118. 33753506 10.1073/pnas.2024288118 PMC8020778

[13] V. A. Kudryavtseva , E. A. Kuzmin , A. V. Moiseeva , et al., “Molecular and Morphological Markers of Neuronal Death in Acute Cerebrovascular Accidents,” Sechenov Medical Journal 13, no. 4 (2022): 18–32.

[14] N. G. Frangogiannis , “Pathophysiology of Myocardial Infarction,” Comprehensive Physiology 5, no. 4 (2015): 1841–1875. 26426469 10.1002/cphy.c150006

[15] P. Yu , S. Ma , X. Dai , and F. Cao , “Elabela Alleviates Myocardial Ischemia Reperfusion‐Induced Apoptosis, Fibrosis and Mitochondrial Dysfunction Through PI3K/AKT Signaling,” American Journal of Translational Research 12, no. 8 (2020): 4467–4477. 32913520 PMC7476165

[16] W. Chen , L. Lv , Z. Nong , X. Chen , X. Pan , and C. Chen , “Hyperbaric Oxygen Protects Against Myocardial Ischemia‐Reperfusion Injury Through Inhibiting Mitochondria Dysfunction and Autophagy,” Molecular Medicine Reports 22, no. 5 (2020): 4254–4264. 32901878 10.3892/mmr.2020.11497 PMC7533464

[17] S. D. Visonà , D. Benati , M. C. Monti , et al., “Diagnosis of Sudden Cardiac Death due to Early Myocardial Ischemia: An Ultrastructural and Immunohistochemical Study,” European Journal of Histochemistry 62, no. 2 (2018): 2866. 29943950 10.4081/ejh.2018.2866 PMC6038110

[18] R. Bagur , S. Tanguy , S. Foriel , et al., “The Impact of Cardiac Ischemia/Reperfusion on the Mitochondria‐Cytoskeleton Interactions,” Biochimica et Biophysica Acta 1862, no. 6 (2016): 1159–1171. 26976332 10.1016/j.bbadis.2016.03.009

[19] A. V. Kuznetsov , S. Javadov , R. Margreiter , M. Grimm , J. Hagenbuchner , and M. J. Ausserlechner , “The Role of Mitochondria in the Mechanisms of Cardiac Ischemia‐Reperfusion Injury,” Antioxidants 8, no. 10 (2019): 454. 31590423 10.3390/antiox8100454 PMC6826663

[20] P. Pal , B. Bhattacharjee , A. K. Ghosh , A. Chattopadhyay , and D. Bandyopadhyay , “Melatonin Protects Against Cardiac Damage Induced by a Combination of High Fat Diet and Isoproterenol Exacerbated Oxidative Stress in Male Wistar Rats,” Melatonin Research 1 (2018): 109–131.

[21] D. P. Del Re , D. Amgalan , A. Linkermann , Q. Liu , and R. N. Kitsis , “Fundamental Mechanisms of Regulated Cell Death and Implications for Heart Disease,” Physiological Reviews 99, no. 4 (2019): 1765–1817. 31364924 10.1152/physrev.00022.2018 PMC6890986

[22] Y. Xiaokaiti and X. Li , “Natural Product Regulates Autophagy in Cancer. In Autophagy: Biology and Diseases,” in Autophagy: Biology and Diseases, ed. W. Le (Springer Singapore, 2020), 709–724. 10.1007/978-981-15-4272-5_53 32671788

[23] L. Jian , Y. Lu , S. Lu , and C. Lu , “Chemical Chaperone 4‐Phenylbutyric Acid Reduces Cardiac Ischemia/Reperfusion Injury by Alleviating Endoplasmic Reticulum Stress and Oxidative Stress,” Medical Science Monitor 22 (2016): 5218–5227. 28036323 10.12659/MSM.898623 PMC5221419

[24] W. K. Zhao , Y. Zhou , T. T. Xu , and Q. Wu , “Perfusion Injury,” Oxidative Medicine and Cellular Longevity 2021 (2021): 9929687. 34725566 10.1155/2021/9929687 PMC8557044

[25] W. Li , W. Li , Y. Leng , Y. Xiong , and Z. Xia , “Ferroptosis Is Involved in Diabetes Myocardial Ischemia/Reperfusion Injury Through Endoplasmic Reticulum Stress,” DNA and Cell Biology 39, no. 2 (2020): 210–225. 31809190 10.1089/dna.2019.5097

[26] L. M. Nepomnyashchikh and V. G. Zimmermann , “Pre‐Necrotic Contracture Damage in Cardiomyocytes: Photochemical Fluorochrome Staining and Fluorescent Microscopy of the Myocardium,” Bulletin of Experimental Biology and Medicine 132, no. 3 (2001): 907–913. 11740591 10.1023/a:1013147524226

[27] H. M. Piper , K. Meuter , and C. Schäfer , “Cellular Mechanisms of Ischemia‐Reperfusion Injury,” Annals of Thoracic Surgery 75, no. 2 (2003): S644–S648. 12607706 10.1016/s0003-4975(02)04686-6

[28] V. G. Zimmermann and L. M. Nepomnyashchikh , “Lytic and Contracture‐Lytic Pre‐Necrotic Damage to Cardiomyocytes: Photochemical Fluorochrome Staining and Fluorescent Microscopy of the Myocardium,” Bulletin of Experimental Biology and Medicine 134 (2002): 571–577. 12660842 10.1023/a:1022969313879

[29] L. M. Nepomnyashchikh , “Principal Forms of Acute Damage to Cardiomyocytes According to Polarization Microscopy Data,” Bulletin of Experimental Biology and Medicine 121 (1996): 1–9.

[30] L. Zhang , C. Q. Xu , Y. Hong , et al., “Propranolol Regulates Cardiac Transient Outward Potassium Channel in Rat Myocardium via cAMP/PKA After Short‐Term but Not After Long‐Term Ischemia,” Naunyn‐Schmiedeberg's Archives of Pharmacology 382 (2010): 63–71. 20499050 10.1007/s00210-010-0520-y

[31] C. J. Pitarys, 2nd , R. Virmani , H. D. Vildibill, Jr. , E. K. Jackson , and M. B. Forman , “Reduction of Myocardial Reperfusion Injury by Intravenous Adenosine Administered During the Early Reperfusion Period,” Circulation 83, no. 1 (1991): 237–247. 1984882 10.1161/01.cir.83.1.237

[32] J. T. Lie , K. E. Holley , W. R. Kampa , and J. L. Titus , “New Histochemical Method for Morphologic Diagnosis of Early Stages of Myocardial Ischemia,” Mayo Clinic Proceedings 46, no. 5 (1971): 319–327. 4102906

[33] C. Pasqualin , F. Gannier , A. Yu , C. O. Malécot , P. Bredeloux , and V. Maupoil , “SarcOptiM for ImageJ: High‐Frequency Online Sarcomere Length Computing on Stimulated Cardiomyocytes,” American Journal of Physiology, Cell Physiology 311, no. 2 (2016): C277–C283. 27335170 10.1152/ajpcell.00094.2016

[34] P. Peterson , M. Kalda , and M. Vendelin , “Real‐Time Determination of Sarcomere Length of a Single Cardiomyocyte During Contraction,” American Journal of Physiology, Cell Physiology 304, no. 6 (2013): C519–C531. 23255581 10.1152/ajpcell.00032.2012 PMC3671565

[35] C. N. Toepfer , A. Sharma , M. Cicconet , et al., “SarcTrack,” Circulation Research 124, no. 8 (2019): 1172–1183. 30700234 10.1161/CIRCRESAHA.118.314505 PMC6485312

[36] D. Tamborrini , Z. Wang , T. Wagner , et al., “Structure of the Native Myosin Filament in the Relaxed Cardiac Sarcomere,” Nature 623 (2023): 863–871. 37914933 10.1038/s41586-023-06690-5 PMC10665186

[37] I. D. Shperling , “The Relaxation of Sarcomeres in Ischemic Injury of Myocardium,” Virchows Archiv. A, Pathological Anatomy and Histology 380, no. 2 (1978): 149–154. 153033 10.1007/BF00430621

[38] L. Lu , Y. Xu , C. R. Greyson , P. C. Ursell , and G. G. Schwartz , “Non Elastic Deformation of Myocardium in Low‐Flow Ischemia and Reperfusion: Ultrastructure‐Function Relations,” Journal of Molecular and Cellular Cardiology 31, no. 5 (1999): 1157–1169. 10336853 10.1006/jmcc.1999.0948

[39] M. Arteaga , C. M. Warren , S. Milutinovic , A. F. Martin , and R. J. Solaro , “Specific Enhancement of Sarcomeric Response to Ca2+ Protects Murine Myocardium Against Ischemia‐Reperfusion Dysfunction,” American Journal of Physiology, Heart and Circulatory Physiology 289, no. 5 (2005): H2183–H2192. 16024565 10.1152/ajpheart.00520.2005

[40] H. Lemcke , A. Skorska , C. I. Lang , L. Johann , and R. David , “Quantitative Evaluation of the Sarcomere Network of Human hiPSC‐Derived Cardiomyocytes Using Single‐Molecule Localization Microscopy,” International Journal of Molecular Sciences 21, no. 8 (2020): 2819. 32316650 10.3390/ijms21082819 PMC7216082

[41] S. Sinha , Y. Elbaz‐Alon , and O. Avinoam , “Ca as a Coordinator of Skeletal Muscle Differentiation, Fusion and Contraction,” FEBS Journal 289, no. 21 (2022): 6531–6542. 35689496 10.1111/febs.16552 PMC9795905

[42] A. Aghigh , S. Bancelin , M. Rivard , M. Pinsard , H. Ibrahim , and F. Légaré , “Second Harmonic Generation Microscopy: A Powerful Tool for Bio‐Imaging,” Biophysical Reviews 15, no. 1 (2023): 43–70. 36909955 10.1007/s12551-022-01041-6 PMC9995455

[43] Y. Pritchard , A. Sharma , C. Clarkin , H. Ogden , S. Mahajan , and R. J. Sánchez‐García , “Persistent Homology Analysis Distinguishes Pathological Bone Microstructure in Non‐Linear Microscopy Images,” Scientific Reports 13 (2023): 2522. 36781895 10.1038/s41598-023-28985-3 PMC9925777

[44] S. V. Plotnikov , A. C. Millard , P. J. Campagnola , and W. A. Mohler , “Characterization of the Myosin‐Based Source for Second‐Harmonic Generation From Muscle Sarcomeres,” Biophysical Journal 90, no. 2 (2006): 693–703. 16258040 10.1529/biophysj.105.071555 PMC1367074

[45] R. Gauderon , P. B. Lukins , and C. J. Sheppard , “Optimization of Second‐Harmonic Generation Microscopy,” Micron 32, no. 7 (2001): 691–700. 11334739 10.1016/s0968-4328(00)00066-4

[46] O. Lookin , P. de Tombe , N. Boulali , C. Gergely , T. Cloitre , and O. Cazorla , “Cardiomyocyte Sarcomere Length Variability: Membrane Fluorescence Versus Second Harmonic Generation Myosin Imaging,” Journal of General Physiology 155, no. 4 (2023): e202213289. 36695814 10.1085/jgp.202213289 PMC9930136

[47] T. Boulesteix , E. Beaurepaire , M. P. Sauviat , and M. C. Schanne‐Klein , “Second‐Harmonic Microscopy of Unstained Living Cardiac Myocytes: Measurements of Sarcomere Length With 20‐Nm Accuracy,” Optics Letters 29, no. 17 (2004): 2031–2033. 15455770 10.1364/ol.29.002031

[48] Y. Tarakanchikova , O. Stelmashchuk , E. Seryogina , et al., “Allocation of Rhodamine‐Loaded Nanocapsules From Blood Circulatory System to Adjacent Tissues Assessed In Vivo by Fluorescence Spectroscopy,” Laser Physics Letters 15 (2018): 105601.

[49] C. Basso , B. Aguilera , J. Banner , et al., “Guidelines for Autopsy Investigation of Sudden Cardiac Death: 2017 Update From the Association for European Cardiovascular Pathology,” Virchows Archiv 471, no. 6 (2017): 691–705. 28889247 10.1007/s00428-017-2221-0 PMC5711979

[50] E. Martínez‐Barrios , S. Grassi , M. Brión , et al., “Molecular Autopsy: Twenty Years of Post‐Mortem Diagnosis in Sudden Cardiac Death,” Frontiers in Medicine 10, no. 10 (2023): 1118585. 36844202 10.3389/fmed.2023.1118585 PMC9950119

[51] G. Piavchenko , A. Alekseev , O. Stelmashchuk , et al., “A Complex Morphofunctional Approach for Zinc Toxicity Evaluation in Rats,” Heliyon 6, no. 4 (2020): e03768. 32337380 10.1016/j.heliyon.2020.e03768 PMC7177034

[52] C. P. Campobasso , A. S. Dell'Erba , A. Addante , F. Zotti , A. Marzullo , and M. F. Colonna , “Sudden Cardiac Death and Myocardial Ischemia Indicators: A Comparative Study of Four Immunohistochemical Markers,” American Journal of Forensic Medicine and Pathology 29, no. 2 (2008): 154–161. 18520484 10.1097/PAF.0b013e318177eab7

[53] G. M. Bürgisser , D. M. Heuberger , P. Giovanoli , M. Calcagni , and J. Buschmann , “Delineation of the Healthy Rabbit Heart by Immunohistochemistry—A Technical Note,” Acta Histochemica 125, no. 1 (2023): 151993. 36584538 10.1016/j.acthis.2022.151993

[54] J. D. Morris and C. K. Payne , “Microscopy and Cell Biology: New Methods and New Questions,” Annual Review of Physical Chemistry 70 (2019): 199–218. 10.1146/annurev-physchem-042018-052527 30883272

[55] K. L. Sloots , J. Vinten‐Johansen , and G. P. Dobson , “Warm Nondepolarizing Adenosine and Lidocaine Cardioplegia: Continuous Versus Intermittent Delivery,” Journal of Thoracic and Cardiovascular Surgery 133, no. 5 (2007): 1171–1178. 17467425 10.1016/j.jtcvs.2006.12.058

[56] B. Mets , P. K. Janicki , M. F. James , R. Erskine , and B. Sasman , “Lidocaine and Bupivacaine Cardiorespiratory Toxicity Is Additive: A Study in Rats,” Anesthesia and Analgesia 75, no. 4 (1992): 611–614. 1530175 10.1213/00000539-199210000-00026

[57] D. Testelmans , K. Maes , P. Wouters , S. K. Powers , M. Decramer , and G. Gayan‐Ramirez , “Infusions of Rocuronium and Cisatracurium Exert Different Effects on Rat Diaphragm Function,” Intensive Care Medicine 33, no. 5 (2007): 872–879. 17361387 10.1007/s00134-007-0584-4

